# Genotoxic effect of exposure to polycyclic aromatic hydrocarbons (PAHs) in asphalt workers

**DOI:** 10.17179/excli2021-3487

**Published:** 2021-03-16

**Authors:** Fatemeh Kargar-Shouroki, Mohammad Miri, Mohammad Javad Zare Sakhvidi, Seyedeh Zahra Hosseini Sangchi, Farzan Madadizadeh

**Affiliations:** 1 Occupational Health Research Center, Department of Occupational Health Engineering, School of Public Health, Shahid Sadoughi University of Medical Sciences, Yazd, Iran; 2 Non-Communicable Diseases Research Center, Department of Environmental Health, School of Health, Sabzevar University of Medical Sciences, Sabzevar, Iran; 3 Occupational Health Research Center, School of Public Health, Shahid Sadoughi University of Medical Sciences, Yazd, Iran; 4 Research Center of Prevention and Epidemiology of Non-Communicable Disease, Department of Biostatistics and Epidemiology, School of Public Health, Shahid Sadoughi University of Medical Sciences, Yazd, Iran

**Keywords:** polycyclic aromatic hydrocarbons (PAHs), micronucleus, asphalt workers, genotoxic effect

## Abstract

Asphalt workers are at risk due to exposure to asphalt fumes containing polycyclic aromatic hydrocarbons (PAHs). The main purpose of this study was to measure the urinary metabolite of PAHs and to determine its effect on micronucleus (MN) formation as an indicator of genotoxic damage. In this cross-sectional study, the MN frequency in 48 male asphalt workers exposed to PAHs was measured and compared with 48 male non-exposed employees. PAHs exposure was evaluated by determining urinary 1-Hydroxypyrene (1-OHP). The mean concentrations of 1-OHP in the exposed and non-exposed groups were 0.58 ± 0.41 μmol/mol creatinine and 0.38 ± 0.25 μmol/mol creatinine, respectively. 1-OHP concentration was significantly higher in smokers compared with non-smokers in both exposed and non-exposed groups. Moreover, the mean MN frequency in the exposed group was significantly higher than in the non-exposed group. The MN frequency was significantly higher in asphalt workers with a work history of ≥ 15 years compared to workers with lower work history. In a fully adjusted model, there was a statistically significant association between exposure to PAHs, with MN and 1-OHP concentration, and between smoking status with 1-OHP. The findings of the present study indicated that occupational exposure to PAHs was associated with increased urinary 1-OHP as well as DNA damage in the asphalt workers.

## Introduction

The polycyclic aromatic hydrocarbons (PAHs), are a ubiquitous class of environmental contaminants. They enter the environment by burning fossil fuels and forest fires (Sram et al., 2016[36]). The general population is exposed to PAHs through the air, food, as well as from cigarette smoke (Bal et al., 2018[3]). Occupational exposures to these compounds occur in asphalt producing, road paving, roofing, coke plants, aluminum plants, and waterproofing operations by inhalation and dermal absorption (Hong and Lee, 1999[20]; Muñoz and Albores, 2011[31]; Jacob and Seidel, 2002[21]). Asphalt is consisting of about 4 % to 5 % of bitumen, as a binder that is heated and mixed with stone and sand. Filler or fibers and aliphatic amines are also added to increase binding and the quality of the asphalt (Ulvestad et al., 2007[39]). 

"Bitumen" itself is formed as a by-product from the heavy residues of crude oil distillation and consists of cyclic alkanes, the heterocyclic compounds containing nitrogen, oxygen, and sulfur, aromatic hydrocarbons, aliphatic compounds, volatile organic compounds (VOCs), metals, and polycyclic aromatic hydrocarbons (PAHs) including benzo [a] pyrene, benzo [a] anthracene, benzo [b]-fluoranthene, chrysene, benzo [k] fluoranthene, indeno [1,2,3-cd] pyrene, dibenzo [ah] anthracene, and benzo [ghi] perylene (Welge et al., 2011[41]; Gaikwad et al., 2020[18]). The potential carcinogenicity of bitumen has been attributed to the presence of PAHs (Marczynski et al., 2011[30]). Recently, the International Agency for Research on Cancer (IARC) classified occupational exposures to asphalt and its emissions during road paving as possibly carcinogenic to humans (Group 2B) primarily due to its PAHs content (Xu et al., 2018[42]). Benzo [a] pyrene, phenanthrene, naphthalene, and chrysene have been found to cause lung, stomach, bladder, leukemia, and skin cancer (Deng et al., 2014[14]; Bal et al., 2018[3]; Çelik et al., 2013[12]; Xu et al., 2018[42]). 

However, there is no general agreement about the genotoxic effects of these compounds and the experimental data are rather controversial (Marczynski et al., 2006[28]; Çelik et al., 2013[12]).

Some studies suggested that exposure to PAHs among asphalt workers is associated with increased MN formation (Arul, 2017[1]; Çelik et al., 2013[12]; Karaman and Pirim, 2009[25]; Lindberg et al., 2008[27]; Serdar et al., 2012[34]; Toraason et al., 2001[37]). However, others have not found a link between exposure to asphalt fumes and an increase in DNA damage in the asphalt workers (Cavallo et al., 2009[11]; Welge et al., 2011[41]; Järvholm et al., 1999[22]; Carstensen et al., 1999[10]). 

Among several cytogenetic methods for assessing genetic damage such as chromosomal aberrations, micronucleus (MN) formation, and sister chromatid exchanges (SCE) (Bacaksiz et al., 2014[2]), micronucleus (MN) assay in peripheral blood lymphocytes is a simple, inexpensive, not invasive and rapid for assessing DNA damage (Cavallo et al., 2009[11]; Sram et al., 2016[36]). Micronucleus is a membrane-bound structure that contains chromosome breaks lacking centromeres (acentric fragments) and/or whole chromosomes that is unable to incorporate into a daughter nucleus at anaphase/telophase and resides in the cytoplasm (Fenech, 2008[17]). The International Human Micronucleus (HUMN) Project of 5424 subjects from 30 laboratories worldwide showed an increase in total cancer risk with increasing MN frequency (Bonassi et al., 2011[4]).

Because of the complexity in the PAHs composition, no established TLVs have been proposed by ACGIH for PAHs (Tsai et al., 2004[38]). However, the PAHs metabolites excreted in the urine are directly related to the adverse health effects of exposure to PAHs because they reflect the amount of toxicant absorbed by all exposure routes (Jacob and Seidel, 2002[21]). Urinary 1-hydroxypyrene (1-OHP) as the main metabolite of pyrene is widely used as an indicator for exposure assessment to PAHs (Xu et al., 2018[42]). Since pyrene is always present in PAH mixtures, 1-OHP is not only an indicator of uptake of pyrene but also an indirect indicator of total exposure to PAHs (Jongeneelen, 2001[24]).

To the best of our knowledge, this is the first study in the literature regarding the determination of DNA damage on Iranian asphalt workers using the MN formation. This study aims to assess urinary 1-OHP and to determine its effect on micronucleus (MN) formation as an indicator of genotoxic damage.

## Material and Methods

### Population settings

The study was performed on 48 male asphalt workers from the Yazd province (Iran) (24 smokers and 24 non-smokers) with a mean age of 39.17±9.14 years and a work history of 13.06±6.40 years. 48 male employees without exposure to PAHs were selected as a non-exposed group (mean age and work history 37.71±6.72 years and 12.08±5.51 years, respectively, 14 smokers and 34 non-smokers). The participants with a medical and occupational history of exposure to other genotoxic agents and those who consume antibiotics and antioxidants (vitamins C, and E) during the last 3 months were excluded from the study. Demographic, medical, and occupational data, including age, work history, height, weight, personnel protective equipment usage, ventilation system, personal habits (smoking status, dietary habit), and diseases, were obtained from a self-administered questionnaire. The study was approved by the Ethics Committee of the Shahid Sadoughi University of Medical Sciences (IR.SSU.SPH.REC.1398.093) and was conducted in accordance with the principles for human experience as defined by the Helsinki Declaration. All subjects gave informed consent before sampling.

### Biological monitoring

20 - 50 ml of the urine samples were collected from participants at the end of the work shift on the weekend, transported to the laboratory, and stored at −20 °C until analysis.

For the preparation of urine samples, solid-phase extraction (SPE) was used according to Jongeneelen and Brucker's methods (Jongeneelen et al., 1987[23]; Brucker et al., 2013[7]). Briefly, 2.5 ml of urine samples were adjusted to pH 5.0, with adding 5 ml of acetate buffer and 10 μl of glucuronidase arylsulphatase to the samples. Then, they were incubated at 37 °C for 2 hrs. The SPE was performed with a C18 cartridge (CHROMABAND® C18ec 3 ml, 500 mg, 50/pk). For activating the C18 cartridge, it was washed with 2 ml methanol and 5 ml distilled water. The prepared sample was then passed through the sorbent at a flow rate of 10 ml/min. The column was then washed out with 6 ml of 40 % methanol. The retained analyte was eluted by 2 ml isopropanol. Finally, the solvent was evaporated at 37 °C and reconstituted with 200 μl methanol.

Measurement of 1-OHP concentration was performed according to Chen's method (Chen et al., 1999[13]). The high-performance liquid chromatography (HPLC) was used to determine 1-OHP concentrations. The HPLC was equipped with a C18 reversed-phase column (250 × 4.6 mm, with 5 μm particle size; Germany). The mobile phase was a mixture of 65 % distilled water and 35 % acetonitrile at a flow rate of 10 ml/min. A fluorescence detector was adjusted at an excitation wavelength of 242 nm and an emission wavelength of 388 nm. The limit of quantification (LOQ) and the limit of detection (LOD) for 1-OHP were 10 ng/L and 3 ng/L, respectively. The measured concentrations of 1-OHP were corrected in terms of µmol/mol creatinine.

### Genotoxic assays

5 ml of the blood samples were collected into tubes containing heparin-lithium as an anticoagulant. The MN test was performed according to Fenech protocol (Fenech, 2007[16]). About 0.5 ml of the blood sample was added to 4.5 ml of RPMI 1640 (culture medium), 15 % fetal calf serum, 1 % antibiotics (penicillin-streptomycin), and 0.1 ml PHA for the final concentration of 5 μg/ml. Then, they were incubated at 37 °C for 72 hrs. Forty-four hours after PHA stimulation, cytochalasin B at a final concentration of 6 μg/ml was added to each culture. Twenty-eight hours after adding Cyt-B, cells were centrifuged at 1200 rpm for 10 min, treated with 10 ml of 0.075 M, KCL hypotonic solution to lyse red blood cells, centrifuged at 1200 rpm for 10 min, fixed in methanol: acetic acid (3:1, v/v), centrifuged again at 1200 rpm for 10 min and finally placed on slides and allowed to dry for 10 min at room temperature. Then cells were stained with 15 % Giemsa for 10 min and coded for blind scoring. The micronucleus frequency in 1000 binucleated (BN) cells was counted using an Olympus CX41 microscope with an ×40 objective. 

### Statistical analyses

Statistical analysis was done through SPSS (version 21, SPSS Inc., Chicago, IL, USA). The quantitative results were expressed as means ± standard deviation (SD). An independent sample t-test was used to determine differences between exposed and non-exposed groups for age, work history, and BMI, and to compare urinary 1-OHP between groups. 

The chi-square test was used to assess the distribution of smoking status between the exposed and non-exposed groups.

The effect of exposure to PAHs, age, work history, BMI, and smoking status on the MN frequency was determined using univariate Poisson regression analysis. To adjust the effects of confounders, the multivariate Poisson regression model included group (exposed and non-exposed groups), smoking status, and categorical variables of age, work history, and BMI as fixed factors. The non-exposed group, non-smokers, age ≤ 35 years, work history < 15 years, and BMI < 25 kg/m^2^ served as a reference group in categorical variables. The incidence rate ratio (IRR) and its 95 % confidence interval (95 % CI) were estimated. Multiple linear regression analysis was used to control the effects of confounding variables on urinary 1-OHP. The correlations between MN frequency and other variables were determined by Pearson's correlation analysis. P-value < 0.05 was considered statistically significant. 

## Results

Table 1(Tab. 1) presents some of the main characteristics of the exposed and non-exposed groups. There were no significant differences between the two studied groups for age, work history, BMI index, and smoking status. None of the workers used the mask and none of the asphalt plants had a ventilation system.

The mean concentration of 1-OHP was significantly higher in the asphalt workers than in the non-exposed group (0.58±0.41 μmol /mol creatinine vs. 0.38±0.25 μmol /mol creatinine, p=0.007).

Table 2(Tab. 2) shows 1-OHP concentration by age, work history, and smoking status in the exposed and non-exposed groups.

The 1-OHP concentration of those with age > 35 years and work history ≥ 15 years was higher than those with age ≤ 35 years and work history < 15 years in both exposed and non-exposed groups. However, the difference did not reach statistical significance. The 1-OHP concentration of the smokers was significantly higher than non-smokers in exposed and non-exposed groups.

Employing univariate Poisson regression analysis, a mean MN frequency of 5.42 ± 1.72 observed in the PAHs-exposed group, which was significantly higher than that in the non-exposed group (4.35 ± 1.30) (Table 3(Tab. 3)).

Exposure to PAHs resulted in 24 % increments in the MN frequency in the exposed group compared with the non-exposed group (IRR=1.24, 95 % CI: 1.04-1.49, p=0.001).

**Figure 1:**
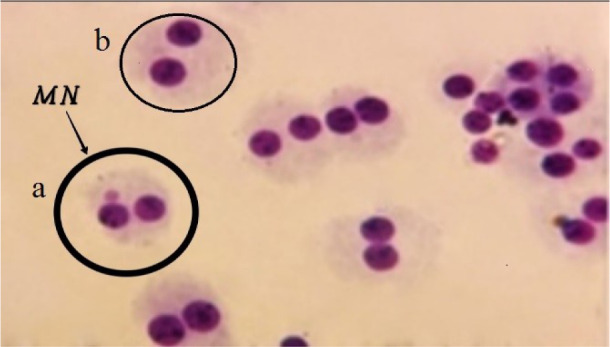
Cell types scored in the MN assay. a: BN cell with a MN, b: BN cell

Examples of BN cells without and with MN are shown in Figure 1(Fig. 1).

Table 4(Tab. 4) shows the MN frequency between exposed and non-exposed groups in terms of age, work history, BMI index, and smoking status. In the exposed group, There was a higher MN frequency in the group over age 35 than in the group under age 35 (IRR = 1.21, 95 % CI: 0.94-1.57, P = 0.14). However, this difference was not statistically significant.

The asphalt workers with a work history of ≥ 15 years had a significantly increased MN frequency compared to those with a work history of < 15 years (IRR = 1.42, 95 % CI: 1.11-1.82, P = 0.006).

The MN frequency in the asphalt workers with BMI of 30-34.99, 25-29.99, and 18.5-24.99 was 5.71 ± 1.60, 5.50 ± 1.90, and 5.28 ± 1.70, respectively. However, the differences were not statistically significant.

There was no significant increase in MN frequency among smoker asphalt workers compared to non-smoker asphalt workers (IRR = 1.24, 95 % CI: 0.97-1.58, p=0.08) (Table 4(Tab. 4)).

No significant difference in MN frequency was found concerning age, work history, and BMI index in the non-exposed group. A mean MN frequency of 4.64 ± 1.90 observed in smokers, which was higher than that in the non-smokers (4.23 ± 0.95) (IRR = 1.10, 95 % CI: 0.82-1.47) (Table 3(Tab. 3)). However, the observed difference was not significant.

The multiple Poisson regression analysis was used to predict the effect of group, age, work history, BMI index, and smoking status on MN frequency. As shown in Table 5(Tab. 5), exposed groups had significantly higher MN frequency than the non-exposed group. No significant association was noted between MN frequency with age, work history, BMI index, and smoking status.

Multiple linear regression models were used to assess the effects of confounding variables of the exposure to PAHs, age, work history, BMI, and smoking status on 1-OHP concentrations (Table 6(Tab. 6)). Results showed that significant positive associations exist between 1-OHP with exposure to PAHs and smoking status after adjusting for confounders.

**Figure 2:**
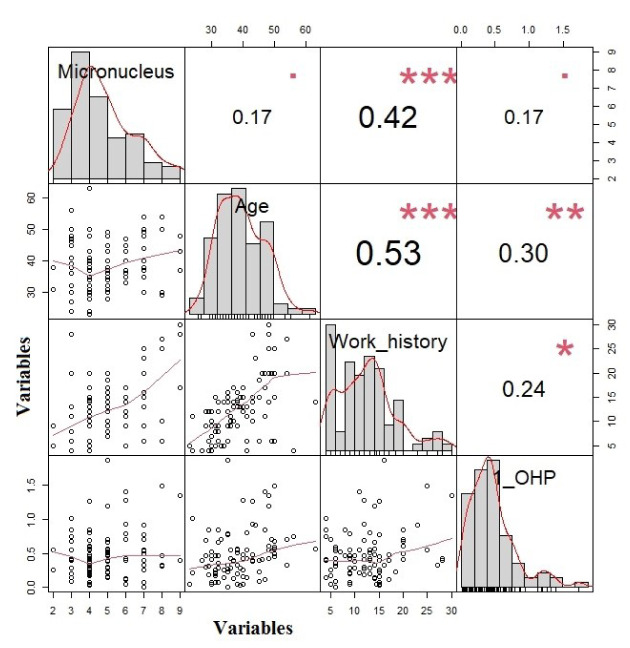
The correlation between micronucleus, 1-OHP, age, and work history (The variables are shown in the columns and the values in each column are the correlation coefficient (r)). *** p<0.001 **p<0.01 * p<0.05

In all subjects a positive correlation between MN frequency and work history was observed (r = 0.42, p < 0.001). 1-OHP was positively correlated with the age, and work history (r = 0.3, p = 0.003; r = 0.24, p = 0.02, respectively) (Figure 2(Fig. 2)).

## Discussion

This study set out to determine the concentration of 1-OHP in the urine of PAHs-exposed and non-exposed groups. The second question in this research was whether (or not) exposure to PAHs is associated with the genotoxic response through MN formation.

Because of the complexity in composition of the PAHs, the threshold limit value (TLV) for total-PAHs has not been established, but the World Health Organization/International Programme on Chemical Safety (WHO/ IPCS) has suggested 0.15-10 µg/m^3^, 20-50 mg/m^3^, and 0.03-0.1 mg/m^3^ for benzo [a] pyrene, naphthalene, and pyrene, respectively (Tsai et al., 2004[38]). 

Boogaard suggested a value of 0.50 μmol/ mol creatinine can be used as an indicator whether or not occupational exposure to PAHs has occurred (Boogaard, 2007[6]). 

Jongeneelen proposed 1-OHP values of 2.3 μmol/mol creatinine (4.4 μg/g) and 4.9 μmol/mol creatinine (9.4 μg/g) are equal to an occupational exposure limit (OEL) of 0.2 mg/m^3^ PAH for coke plant and aluminum industry, respectively. Jongeneelen also suggested 0.24 μmol/mol creatinine and 0.76 μmol/mol creatinine for non-smokers and smokers in non-occupational exposed controls. The authors found no biological effect level of 1-OHP at a concentration of 1.4 μmol/mol creatinine (Jongeneelen, 2001[24]).

In the present study, the mean values of 1-OHP for the exposed and non-exposed groups were 0.58 ± 0.41 μmol/mol creatinine and 0.38 ± 0.25 μmol/mol creatinine, respectively. 

These values are higher than those reported by other investigators. For instance, in Boogaard's study, in the oil workers exposed to bitumen, the mean urinary concentration of 1-OHP was relatively low and ranged between 0.12-0.17 µmol/mol creatinine (Boogaard, 2007[6]).

Xu et al. showed a significant increase at urinary 1-OHP in 167 asphalt workers of Sweden in comparison with a control group (0.076 μmol/mol creatinine (0.021-0.27) vs 0.028 μmol/mol creatinine (0.0092-0.091)) (Xu et al., 2018[42]).

But, Sellappa et al. reported a mean urinary 1-OHP concentration of 1.68 ± 0.93 µmol/mol creatinine in Indian road pavers in comparison to controls (0.55 ± 0.42 µmol/mol creatinine) (Sellappa et al., 2011[33]).

Similarly, Bal et al. reported significantly increased urinary concentrations of 1-OHP in asphalt workers exposed to PAHs in comparison with a control group (1.18 μmol/mol creatinine vs 0.1 μmol/mol creatinine) (Bal et al., 2018[3]).

Different 1-OHP concentrations may be due to differences in the environmental exposures, asphalt composition, application technologies, type of bitumen, and its temperature (Boogaard, 2007[6]; Marczynski et al., 2007[29]). Lifestyle factors such as smoking may have direct or indirect impacts on the level of 1-OHP. In present study, in both exposed and non-exposed groups, higher values of 1-OHP were found in smokers compared to non-smokers. In a similar study in Finland, Vaananen et al. reported the concentration of 1-OHP of 0.66 ± 0.58 µmol /mol creatinine in smoker asphalt workers compared to 0.27 ± 0.15 µmol/mol creatinine in non-smokers (Väänänen et al., 2006[40]). Similar findings were reported by other investigators (Pesch et al., 201[32]1; Campo et al., 2006[9]; Serdar et al., 2012[34]; Heikkilä et al., 2002[19]; Buratti et al., 2007[8]).

Similar to our findings, Marczynski et al. and Pesch et al. reported that age is not associated with 1-OHP concentrations (Pesch et al., 2011[32]; Marczynski et al., 2006[28]). 

In the present study, exposure to PAHs was found to cause an increased MN frequency in asphalt workers in comparison with a non-exposed group.

These findings are consistent with those of Karaman and Pirim who reported a significantly higher MN frequency in PAHs-exposed workers in comparison to the non-exposed group. Urinary concentration of 1-OHP was 0.39 ± 0.21 µmol/mol creatinine vs. 0.16 ± 0.08 µmol/mol creatinine (Karaman and Pirim, 2009[25]).

Similarly, in 2013, Celik et al. in a study on 40 road construction workers of Turkey, reported significantly elevated MN frequency in the exposed group as compared to a control group (Çelik et al., 2013[12]).

Kumar et al. also reported increased MN frequency in road construction workers (7.03 ± 2.08) in comparison to a non-exposed control group (3.35 ± 1.10) (Kumar et al., 2011[26]). Similar results have been reported by Arul (2017[1]). 

However, these results are inconsistent with the findings of Järvholm et al., who found no significant increase in MN frequency in PAHs-exposed workers compared with the non-exposed group (Järvholm et al., 1999[22]).

In 2011, Welge et al. in a study of 225 asphalt workers and 69 control individuals, reported no significant increases in MN frequency of exposed individuals (Welge et al., 2011[41]).

This rather contradictory result may be due to individual differences in toxicant absorption and disposition, environmental exposures, duration and concentration of exposure, statistical analysis, personal protective equipment used, sample size, personal or lifestyle variables, such as BMI, the workload that affects pulmonary ventilation, or cigarette smoking that could affect the metabolic status of an individual (Jacob and Seidel, 2002[21]).

The results of this study did not show any significant increase in MN frequency in older person in comparison to younger persons, that is inconsistent with the findings of Welge et al. (2011[41]) who suggested age is the predictor of MN formation in both bitumen-exposed workers and referents. 

In 2001, Bonassi et al. in a study of 800 subjects (on average) also reported significant increases in MN frequency of individuals with an age of higher than 30 years (Bonassi et al., 2001[5]) and Duan et al. found subjects with age over 44 years have significantly higher MN frequencies (11.36 ± 0.98 %) than those with age less than 35 years (8.58 ± 1.05 %) (Duan et al., 2009[15]). This inconsistency may be due to the small sample size in the present study.

In our study, workers with high work history had significantly higher MN frequency than low work history workers, which is consistent with results reported by Singh et al. who reported a higher MN frequency in the PAHs-exposed workers with more years of work than those with fewer years (Singh et al., 2011[35]). 

Although higher MN frequency was found in smokers compared to non-smokers, the difference was not statistically significant. This result is similar to those reported by some writers (Welge et al., 2011[41]; Çelik et al. 2013[12]; Duan et al., 2009[15]).

Observed effects in the present study, is further supported by the results of regression analysis where significant associations were found between exposure to PAHs, with MN and 1-OHP concentration, and also between smoking with 1-OHP after adjusting for the role of potential confounders of age, work history, BMI, and smoking status.

These findings are also similar to Marczynski's study where higher 1-OHP have been reported in PAHs-exposed workers after adjusting for age, smoking, and nationality (Marczynski et al., 2006[28]).

In the present study, there was a positive correlation between MN frequency and 1-OHP with work history and between 1-OHP with the age. There was no significant correlation between MN frequency and 1-OHP.

In line with our results, in the Turkish study, there was no significant correlation between the urinary 1-OHP concentration and MN frequency (Karaman and Pirim, 2009[25]). Welge et al. also did not find any association between MN frequency and exposure to vapors and aerosols of bitumen (Welge et al., 2011[41]). 

## Conclusion

These findings suggest that exposure to PAHs is associated with an increase of exertion of 1-OHP and genotoxic effects. We also found smoking and work history had a significant effect on 1-OHP levels and MN frequency, respectively. Therefore, engineering controls and using personal protective equipment are needed to reduce workers' exposure to PAHs. 

## Acknowledgements

The authors thank Shahid Sadoughi University of Medical Sciences, Yazd, Iran (grant No. 6720) for funding this study.

## Conflict of interest

The authors declare that they have no conflict of interest.

## Figures and Tables

**Table 1:**
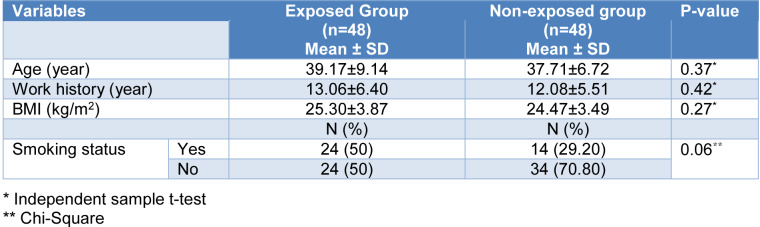
Demographic characteristics of the studied subjects

**Table 2:**
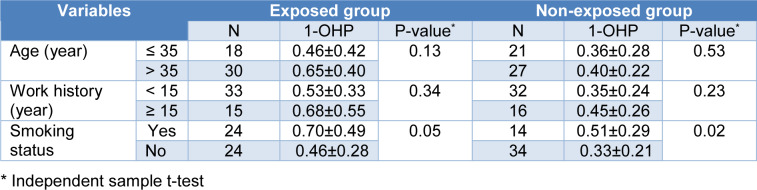
The 1-OHP concentration in the exposed and non-exposed groups by study variables

**Table 3:**
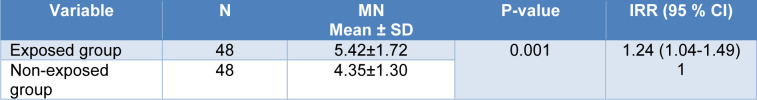
The MNs frequencies in the studied groups

**Table 4:**
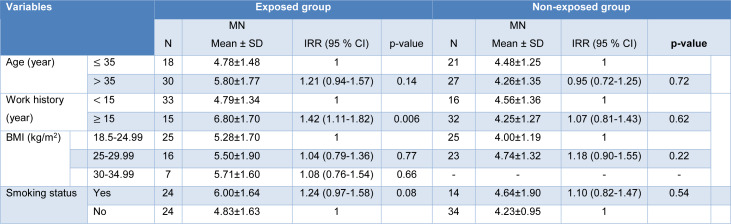
The MN frequencies in the exposed and non-exposed groups by study variables

**Table 5:**
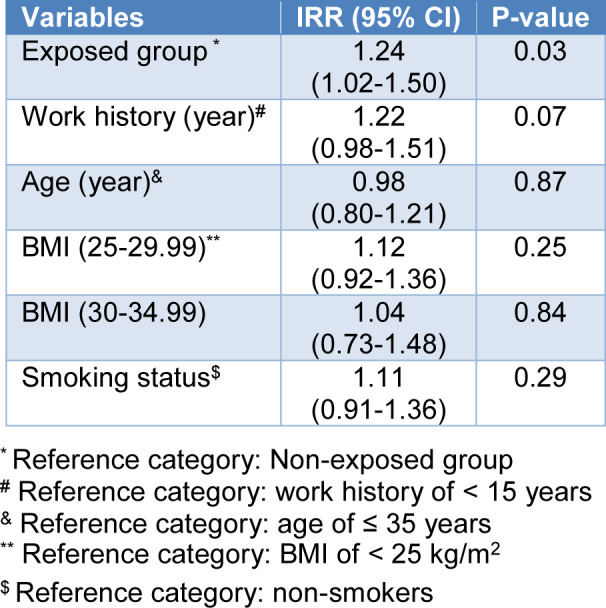
Multiple Poisson regression analysis of the association between independent variables and MN frequencies in the studied groups

**Table 6:**
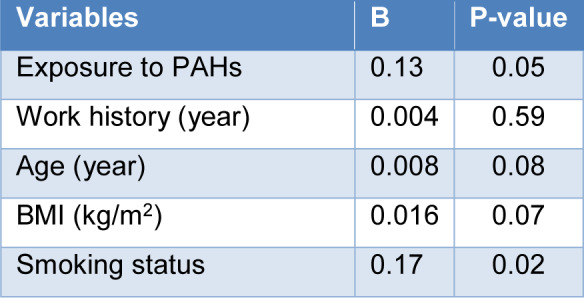
Multiple linear regression analysis of the association between independent variables and 1-OHP in the studied groups
